# The efficacy of different types of intradialytic exercise for patients undergoing hemodialysis: a systematic review and network meta-analysis of randomized controlled trials

**DOI:** 10.1186/s12882-025-04381-z

**Published:** 2025-08-11

**Authors:** HaiQiang Jiang, Yu Wang, Jia Peng, Shuang Wu, Chuanfang Wu

**Affiliations:** 1 School of Nursing, University of South China, Hengyang, 421001 China; 2 Hemodialysis Center, Department of Nephrology, First Affiliated Hospital of Nanchang University, Nanchang, 330000 China; 3 Department of Nursing, Changsha Central Hospital, Affiliated to University of South China, Changsha, 410004 China

**Keywords:** Renal dialysis, Exercise, Dialysis adequacy, Network meta-analysis

## Abstract

**Background:**

Intradialytic exercise interventions improve dialysis efficacy; however, their comparative efficacy remains unclear, limiting evidence-based clinical practice.

**Objective:**

To systematically review and compare the effects of different intradialytic exercises on dialysis adequacy in patients undergoing hemodialysis.

**Methods:**

A systematic search was conducted in PubMed, EMBASE, Web of Science, and the Cochrane Library for randomized controlled trials evaluating the efficacy of intradialytic exercise on dialysis adequacy in patients undergoing hemodialysis. Studies published in the database from its inception to November 1, 2023, were included. Statistical analyses were performed using Stata 15.0.

**Results:**

A total of 24 studies involving 786 patients were included in this analysis. The interventions comprised intradialytic aerobic exercise (IAE), intradialytic resistance exercise (IRE), combined intradialytic aerobic and resistance exercise (IAE + IRE), intradialytic respiratory muscle exercise (IRME), intradialytic electrical muscle stimulation (IEMS), and routine hemodialysis nursing (RHN). Among all pairwise comparisons, only IRME versus RHN showed a statistically significant difference (MD = 0.09, 95% CI: 0.03–0.16). No statistically significant differences were observed in any other pairwise comparisons, including those involving RHN and those between different exercise modalities. Nevertheless, IAE + IRE demonstrated the highest surface under the cumulative ranking curve (SUCRA) values for both the urea clearance index and reduction rate.

**Conclusion:**

Current evidence is insufficient to conclude that any specific type of intradialytic exercise significantly improves dialysis adequacy. Nevertheless, SUCRA rankings indicate a potential benefit, with IAE + IRE demonstrating the highest probability of benefit. Given the limited statistical power, further high-quality studies are warranted to confirm these findings. The review protocol has been registered with PROSPERO(CRD42023484645).

**Clinical trial number:**

Not applicable.

**Supplementary Information:**

The online version contains supplementary material available at 10.1186/s12882-025-04381-z.

## Introduction

Chronic kidney disease (CKD), commonly resulting from hypertension, diabetes, or glomerular diseases [1], affects approximately 85 million people globally and represents a significant public health concern [2]. As CKD progresses to end-stage renal disease, renal replacement therapies become necessary, with hemodialysis being the most widely used modality. By 2030, the number of patients requiring maintenance hemodialysis is projected to reach 5.44 million [3]. 

Adequate dialysis is crucial for enhancing patient outcomes and improving quality of life. It is commonly assessed using urea clearance indicators, such as single-pool Kt/V (spKt/V) and urea reduction ratio (URR), which reflect the efficiency of small solute removal. The KDOQI guidelines state that dialysis adequacy should meet a Kt/V ratio of greater than 1.2 or a URR of greater than 65%[4]. Inadequate dialysis leads to the accumulation of metabolic waste, resulting in acute complications like hyperkalemia and metabolic acidosis [5], and chronic issues including cardiovascular disease, osteoporosis, and neurological disorders [6, 7]. These contribute to a reduced quality of life and increased mortality [8]. 

Several strategies have been explored to improve dialysis adequacy, including extending dialysis duration and increasing the frequency of dialysis [9]. However, poor patient adherence and higher associated costs often limit these approaches, restricting their widespread implementation. Increasing the dialysis blood flow rate has also been proposed as an effective method to enhance adequacy; however, it may be accompanied by adverse effects such as fatigue and shortness of breath [10]. In contrast, Intradialytic exercise performed during hemodialysis is widely considered beneficial for patients [11, 12],. It is conducted under the guidance of healthcare professionals to ensure safety and feasibility [10]. 

Intradialytic exercise holds substantial practical value for clinical practice. Simple equipment, such as dumbbells or recumbent bicycles, benefits patients. Given its feasibility and good tolerance among most hemodialysis patients, intradialytic exercise can be conveniently incorporated into dialysis sessions with appropriate monitoring in place. Healthcare providers play a key role in implementing these interventions by adjusting exercise intensity and duration according to individual patient conditions, thereby minimizing risks and maximizing potential benefits [13]. 

Although intradialytic exercise offers benefits, the comparative effectiveness of various modalities remains uncertain. Common types include aerobic (IAE), resistance (IRE), combined aerobic and resistance (IAE + IRE), respiratory muscle (IRME), and electrical muscle stimulation (IEMS). We conducted a network meta-analysis to integrate direct and indirect evidence, enabling a comprehensive assessment of their relative efficacy. This approach enhances understanding of consistency across studies and provides robust evidence to guide clinical decision-making.

Several network meta-analyses have evaluated the effects of intradialytic exercise on dialysis adequacy; however, methodological differences and inconsistent results persist. One study included nine exercise types, including non-intradialytic exercises [14], 

While non-intradialytic exercise is a viable option, it presents challenges related to self-monitoring and patient safety. Patients adjusting exercise intensity and duration independently may risk under-exercising or over-exercising, potentially leading to adverse health outcomes. Furthermore, if health issues arise, timely intervention may be complex without professional supervision. These concerns highlight the rationale for focusing on intradialytic exercise in our research. Another study examined only four types of intradialytic exercise and used Kt/V as the sole indicator, without considering the URR, which introduces certain limitations [15]. 

To address limitations in existing research, this study systematically evaluates and compares five intradialytic exercise modalities on dialysis adequacy in hemodialysis patients. A network meta-analysis of high-quality randomized controlled trials will be conducted, with Kt/V and URR as primary outcomes. Interventions will be ranked using the surface under the cumulative ranking curve (SUCRA), and consistency between Kt/V and URR rankings will be assessed. If the rankings align, it may indicate that a specific exercise modality offers greater potential benefits in improving dialysis adequacy, thereby providing more substantial evidence to support its clinical application. This will offer a scientific basis for developing clinical exercise intervention programs.

## Methods

### Registration

This systematic review and network meta-analysis were conducted under the Preferred Reporting Items for Systematic Reviews and Meta-Analyses (PRISMA) guidelines [16]. The registration details are available at: https://www.crd.york.ac.uk/PROSPERO/#myprospero.

### Search strategy

A systematic search of PubMed, Embase, Web of Science, and the Cochrane Library was performed to identify studies on the impact of exercise on dialysis adequacy in individuals undergoing hemodialysis. The search spanned the inception of each database until November 1, 2023, using both MeSH and free-text terms. The search terms included renal dialysis, hemodialysis, intermittent hemodialysis, intermittent chronic hemodialysis, dialysis center, chronic intermittent hemodialysis, chronic hemodialysis, blood dialysis, Renal Dialyses, maintenance hemodialysis; exercise, physical workout, physical exertion, physical effort, fitness workout, fitness training, exertion, effort, Physical Activity, Physical Activities; dialysis adequacy, dialysis efficacy, urea reduction ratio, Urea Removal Index, and urea clearance. To ensure comprehensiveness, we also reviewed references from published systematic reviews and meta-analyses for any additional relevant studies. Detailed search strategies for each database are provided in the Supplementary Material, Appendix 1.

### Eligibility criteria

The inclusion criteria for the literature are as follows:①Study Population: Individuals receiving hemodialysis treatment, aged over 18 years.②Intervention: The intervention group receives an exercise intervention during dialysis treatment. including IAE, IRE, IAE + IRE, IRME, or IEMS. ③Control: The control group should receive RHN without structured exercise intervention. ④Primary Outcome Measures: Kt/V and URR. ⑤ Study Type: Randomized controlled trials. Exclusion Criteria ① Studies published in conference abstracts, research proposals, etc. ②Non-English language publications. ③Studies with incomplete or non-extractable data, such as missing key outcome measures (e.g., Kt/V or URR), or data presented in a format that cannot be used for statistical analysis (e.g., missing raw data or data presented in an unstructured format). ④Studies with a quality assessment grade of C.

### Data extraction

Literature management was performed using EndNote 21. Two independent reviewers screened studies and extracted data using the predefined inclusion and exclusion criteria. In the event of any disagreement, a third researcher was consulted to discuss and decide on the inclusion. The extracted data included the first author, publication year, sample size, patient age and gender, type of intervention, exercise details, exercise duration, exercise intensity, exercise frequency, personnel implementing the intervention, and outcome measures (Kt/V or URR).

### Quality assessment

Two reviewers (J.P., S.W.) independently assessed the quality of the included studies using the Cochrane Risk of Bias Tool (version 2.0). The evaluation process encompassed five domains: bias arising from the randomization process, bias due to deviations from intended interventions, bias due to missing outcome data, bias in the measurement of the outcome, and bias in the selection of the reported result. Each domain was assessed as “low risk,” “high risk,” or “unclear risk.” If all domains were rated “low risk,” the study quality was classified as grade (A) If some domains were rated as “unclear risk” and “low risk” with no “high risk” ratings, the study quality was classified as grade (B) If any domain was rated as “high risk,” the study quality was classified as grade C [17]. Disagreements between the two reviewers were resolved through discussion. A third reviewer (C.F.W) was consulted to reach a consensus if necessary.

### Statistical aynthesis and analysis

A meta-analysis was conducted using Stata 15.0 statistical software, with the mean difference (MD) as the effect measure, and 95% confidence intervals (CI) were calculated. A node-splitting model was used to compare direct and indirect comparison results, assessing consistency in the findings. If *P* > 0.05, a consistency model was applied for statistical analysis; if *P* < 0.05, the source of inconsistency was analyzed. An inconsistency test was performed on the closed loops formed by direct and indirect evidence, and the inconsistency factor (IF) was obtained under 95% CI. If the 95% CI includes 0, it indicates a low likelihood of loop inconsistency. A funnel plot was used to assess potential publication bias. The effectiveness of each intervention was ranked using the surface under the cumulative ranking curve (SUCRA); the larger the surface area, the greater the likelihood of benefit. The significance level was set at α = 0.05.

## Results

### Eligible studies

**Fig. 1:**
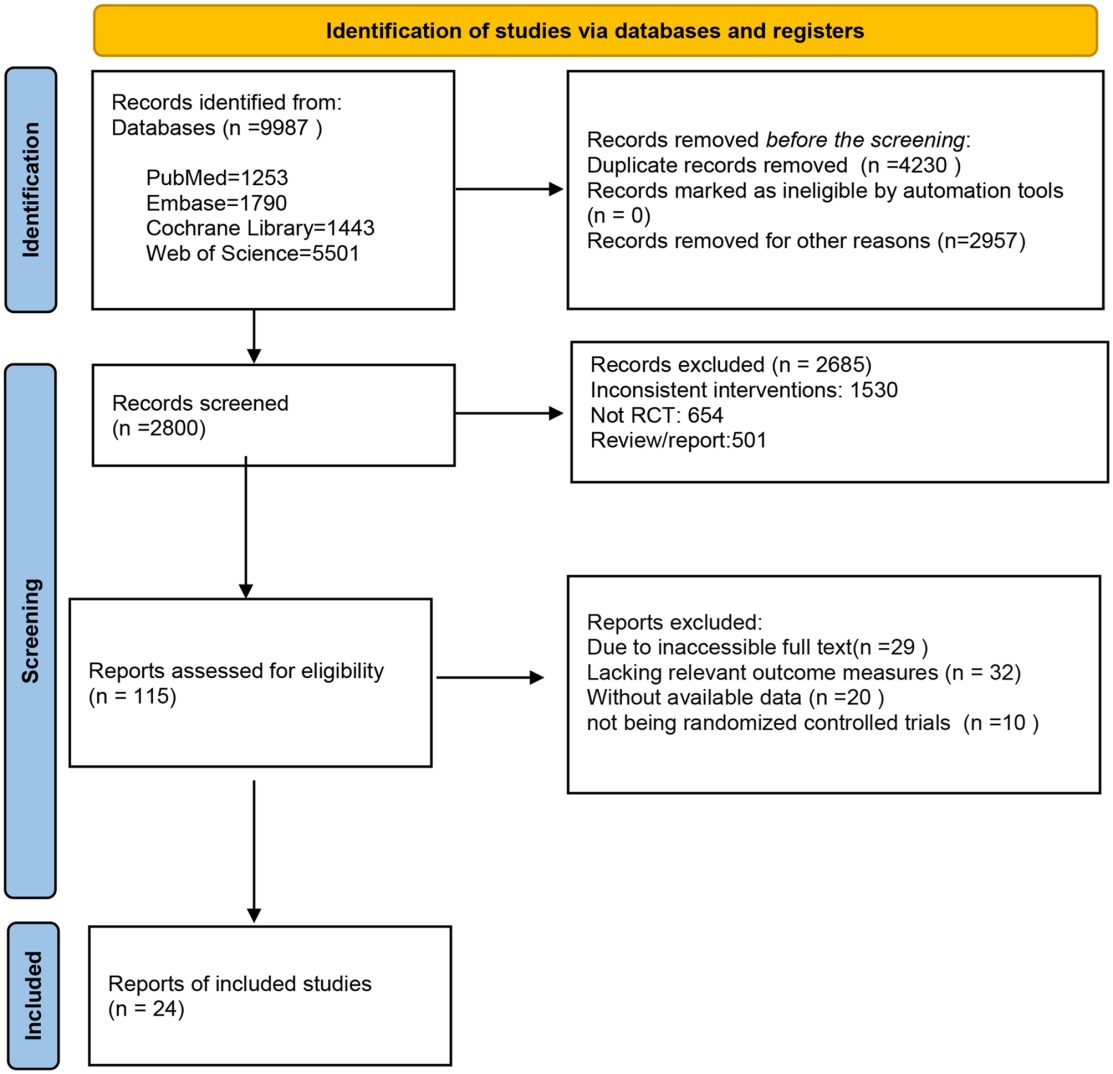
Literature screening flowchart

A systematic search of the Cochrane Library, Embase, PubMed, and Web of Science databases identified 9,987 studies. After removing 4,230 duplicates and 2,957 irrelevant studies, 2,800 additional studies were excluded following abstract review. 115 studies remained for full-text review. Of these, 29 were excluded due to inaccessible full texts, 32 for lack of relevant outcome measures, 20 for unavailable data, and 10 for not being randomized controlled trials. Ultimately, 24 randomized controlled trials were included in the meta-analysis. The study selection process is illustrated in Fig. 1.

### Study characteristics

This study included 24 randomized controlled trials involving 786 patients, covering five types of exercise interventions: IAE [10, 12, 18–31], IRE [32–37], IEMS [12, 38], IRME [32], and IAE + IRE [21, 37, 39]. These 24 studies assessed dialysis adequacy using the Kt/V. And 6 of them used the URR to evaluate dialysis adequacy, involving 226 patients. The four exercise modalities assessed in these studies included IAE [10, 12, 21, 28, 31], IRE [36], IEMS [12], and IAE + IRE [21]. The essential characteristics of the included studies are provided in Supplementary Material Appendix 2.

### Quality of included studies

**Fig. 2:**
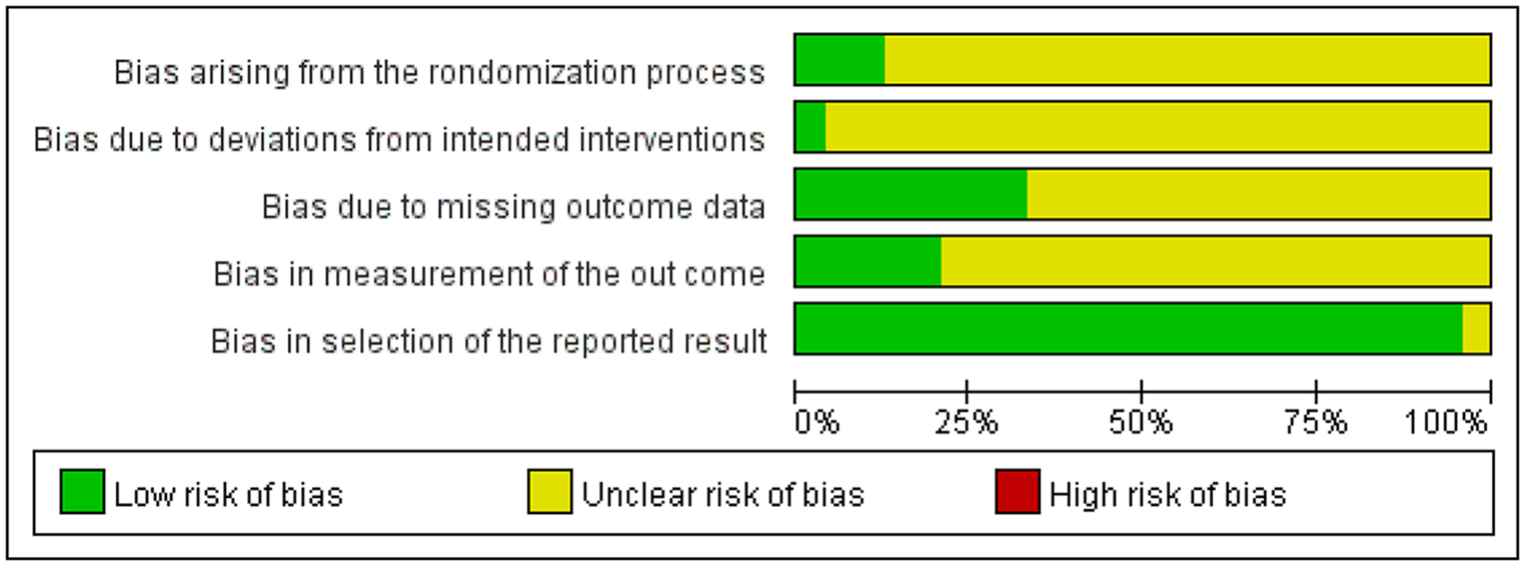
Summary of risk of bias for the included studies

Two researchers assessed the risk of bias in the 24 randomized controlled trials using version 2.0 of the Cochrane Risk of Bias Assessment Tool. All studies were rated with a quality grade of B and included in the network meta-analysis. The risk of bias for each study is summarized in Fig. 2.

### Network meta-analysis results

#### Kt/V

**Fig. 3:**
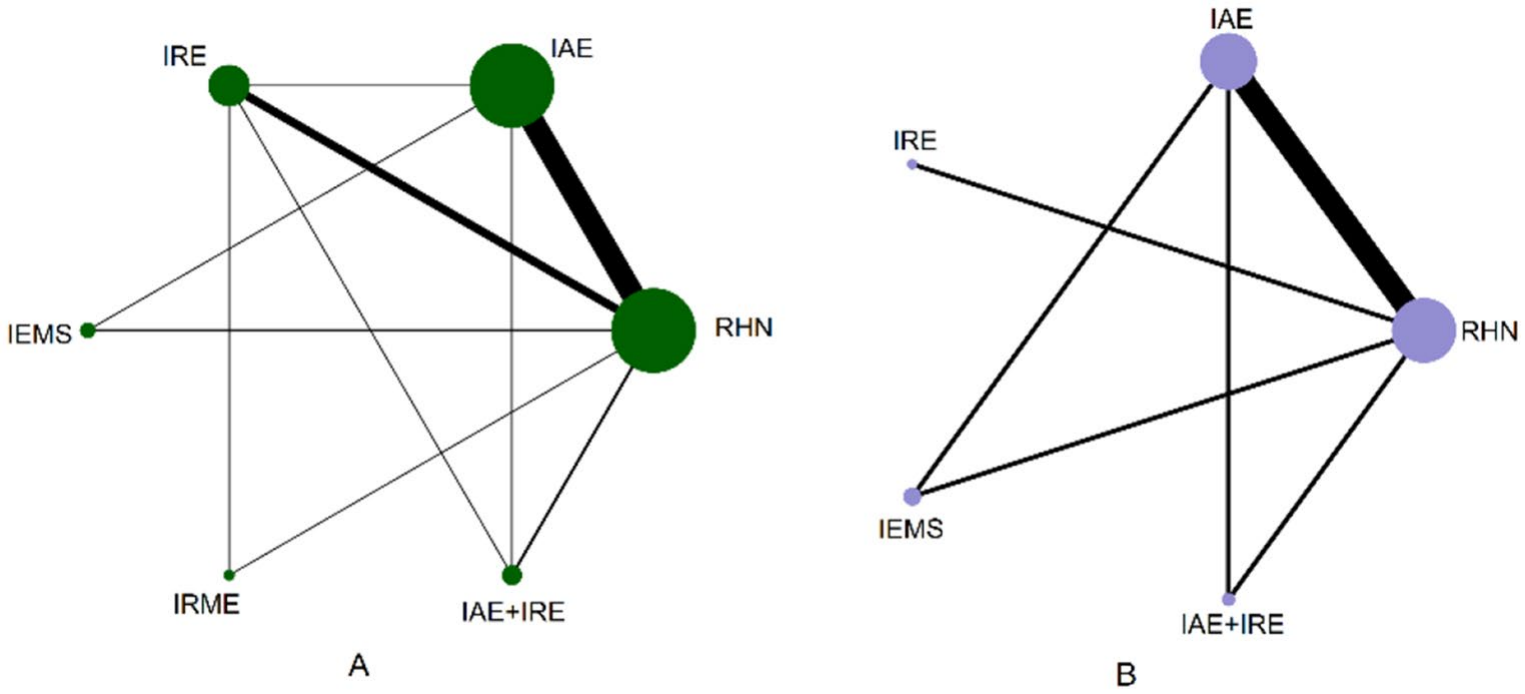
Intradialytic exercise network urea clearance Rate (Kt/V) Network Diagram (**A**) Urea Reduction Ratio (URR) Network Diagram (**B**)

**Fig. 4:**
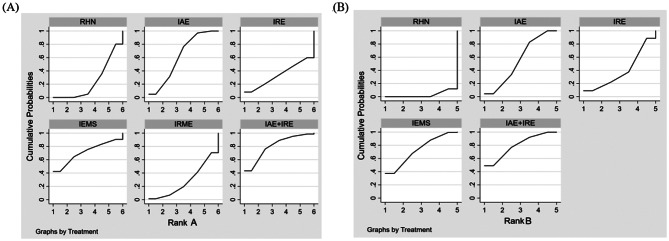
A. SUCRA Rankings of Different Exercise Modalities for Improving Dialysis Adequacy in Hemodialysis Patients (**A**) Kt/V, (**B**) URR

The network diagram of the six closed loops formed by the five exercise modalities(IAE, IRE, IEMS, IRME, and IAE + IRE) compared with RHN is shown in Fig. 3A. Consistency was tested using the node-splitting method, and the results showed P-values greater than 0.05, indicating no significant inconsistency between direct and indirect comparisons. Therefore, a consistency model was used for the analysis. The network meta-analysis results showed that, except for the statistical significance between IRME and RHN (MD = 0.09, 95% CI: 0.03–0.16), no statistically significant differences were observed among other pairwise comparisons (see the lower left of Table 1). Based on SUCRA rankings, the effectiveness of intradialytic exercise interventions in improving Kt/V was ranked as follows: IAE + IRE(80.5)>IEMS 71.3 >IAE(62.0)>IRE(34.0)>IRME(27.9)>RHN(24.2) (Fig. 4A).

**Table 1:** League table of different intradialytic exercise modalities on Dialysis adequacy

IAE + IRE	0.03 (-22.54, 22.60)	-1.21 (-19.76, 17.35)	3.67 (-18.92, 26.26)		3.26 (-12.80, 19.32)
0.01 (-0.40, 0.42)	IEMS	-1.24 (-19.90, 17.43)	3.64 (-19.04, 26.32)		8.14 (-1.44, 17.71)
0.10 (-0.12, 0.32)	0.09 (-0.27, 0.45)	IAE	4.88 (-13.82, 23.57)		-1.84 (-20.43, 16.75)
0.19 (-0.19, 0.57)	0.18 (-0.30, 0.66)	0.09 (-0.23, 0.42)	IRE		-2.43 (-21.05, 16.19)
0.19 (-0.08, 0.46)	0.18 (-0.21, 0.57)	0.09 (-0.08, 0.26)	-0.00 (-0.36, 0.36)	IRME	
0.10 (-0.24, 0.43)	0.07 (-0.16, 0.30)	-0.10 (-0.45, 0.24)	0.08 (-0.25, 0.40)	0.09 (0.03, 0.16)	RHN

Network Meta-analysis of the Effects of Different Exercise Modalities on the Improvement of Urea Clearance Rate (Kt/V) in HD Patients (Mean Difference, MD; 95% Confidence Interval, CI) **Upper right corner**: Network Meta-analysis of the Effects of Different Exercise Modalities on Improving Urea Reduction Ratio (URR) in HD Patients (Mean Difference, MD; 95% Confidence Interval, CI); Intradialytic aerobic exercise (IAE), Intradialytic resistance exercise (IRE), Intradialytic aerobic combined resistance exercise (IAE + IRE), Intradialytic respiratory muscle exercise (IRME), Intradialytic electrical muscle stimulation (IEMS). Routine hemodialysis nursing (RHN)

#### URR

The network diagram of the two closed loops formed by the four exercise modalities (IAE, IRE, IEMS, and IAE + IRE) compared with RHN is shown in Fig. 3B. Consistency was assessed using the node-splitting method, and the results indicated P-values greater than 0.05, suggesting no significant inconsistency between direct and indirect comparisons. Therefore, a consistency model was applied for the analysis. The network meta-analysis results revealed no significant differences among the four types of intradialytic exercise in improving URR in patients undergoing hemodialysis (see the upper right of Table 1). Based on SUCRA rankings, the effectiveness of intradialytic exercise interventions in improving URR was ranked as follows: IAE + IRE (61.9) > IEMS (61.6) > IAE (56.0) > IRE (46.2) > RHN (24.4) (Fig. 4B).

#### Assessment of inconsistency

**Fig. 5:**
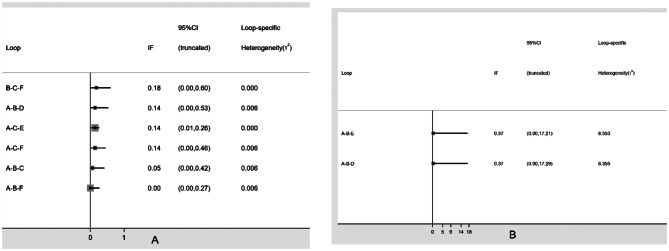
– Loop-specific approach for inconsistency assessment of all comparisons. (**A**) Kt/V, (**B**) URR

Inconsistency tests were performed on the closed loops. For Kt/V, the five exercise modalities formed six closed loops, with the 95% CI of five loops including 0 and the 95% CI of one loop approaching 0 (see Fig. 5A). For URR, the four exercise modalities formed two closed loops, and both loops had 95% CIs that included 0 (see Fig. 5B). The results indicate a low likelihood of inconsistency within the closed loops for Kt/V and URR.

### Publication bias

**Fig. 6:**
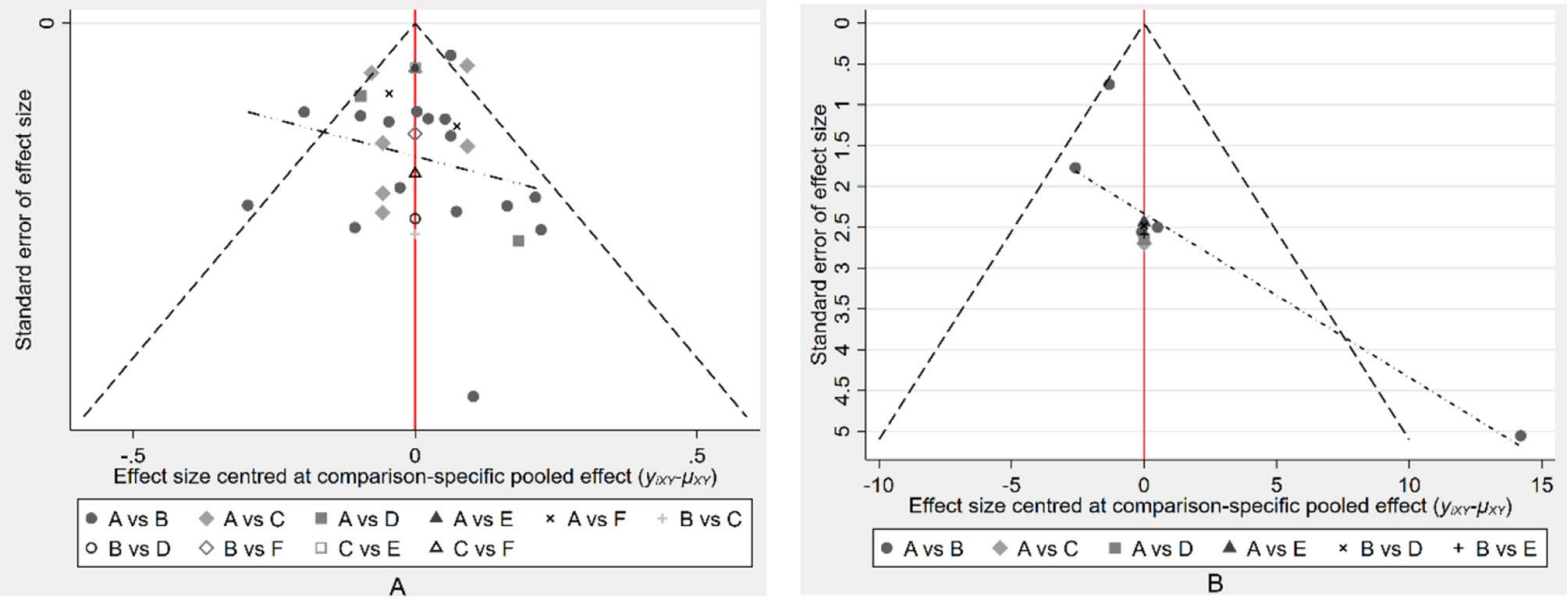
Funnel Plot for Publication Bias Urea Clearance Rate (Kt/V) Publication Bias (**A**) Urea Reduction Ratio (URR) Publication Bias (**B**)

From the funnel plots for the two outcome measures, Kt/V and URR, the studies appear symmetrically distributed on both sides of the funnel plots, indicating a low likelihood of publication bias (see Fig. 6A and B).

## Discussion

In this study, dialysis adequacy was assessed using two key indicators: single-pool Kt/V and urea reduction ratio (URR). A network meta-analysis was employed to evaluate and compare the effects of five different types of intradialytic exercise on these outcomes. The results showed that, among all pairwise comparisons, only IRME versus RHN showed a statistically significant difference (MD = 0.09, 95% CI: 0.03–0.16). No statistically significant differences were observed in any other pairwise comparisons, including those involving RHN and those between different exercise modalities. However, based on the SUCRA rankings for Kt/V and URR, IAE + IRE consistently achieved the highest surface under the cumulative ranking curve values across both indicators. Moreover, the ranking trends of the five exercise types were consistent, suggesting that IAE + IRE may offer greater potential benefits in improving dialysis adequacy.

This study further supports the findings of Ferrari et al., which suggest that IAE + IRE offers the greatest potential benefit in improving dialysis adequacy [40]. The likely physiological mechanism stems from the synergistic effects of both exercise modalities in enhancing dialysis outcomes [21, 41]. Aerobic exercise improves cardiovascular health, enhances heart function, and promotes better blood circulation; It effectively facilitates the transport of oxygen and nutrients while increasing the excretion of waste and toxins, benefiting patients. Resistance exercises enhance muscle strength and endurance, improving the body’s functional capacity and contributing to overall better health. The combination of IAE and IRE comprehensively improves dialysis outcomes across various physiological levels, resulting in a greater likelihood of benefit.

Implementing IAE and IRE requires a certain level of physical strength, often compromised in dialysis patients due to end-stage renal disease, leading to muscle weakness and limb fatigue [42, 43]. Patients with severe motor impairment may be unable to perform these exercises. IEMS, a passive method that stimulates the quadriceps and calf muscles via a portable device, was introduced as a safe and convenient alternative to address this. In the SUCRA ranking, IEMS was more effective than IAE or IRE alone, second only to IAE + IRE, consistent with Song’s systematic review [44]. The reasons for IEMS’s superiority over IAE and IRE remain unclear, but studies in patients with chronic obstructive pulmonary disease and diabetes suggest its effectiveness [45]. Dobsak hypothesized that IEMS, by directly stimulating the muscles, may more effectively improve muscle function and recovery [12]. 

The results of this study differ somewhat from the findings of Katia et al., who concluded that IAE is the most effective exercise modality for improving dialysis adequacy [46]. However, this discrepancy may stem from their study not distinguishing between intradialytic and non-intradialytic exercise. Non-intradialytic exercise refers to physical activities outside the dialysis session, typically in gyms, at home, in parks, or other locations. This categorization may overlook the potential impact of exercise timing on dialysis outcomes, which could account for the differences between their findings and those of our study.

IRME is a novel intradialytic exercise among the various methods explored to improve dialysis adequacy. IRME enhances the strength and endurance of the respiratory muscles by using devices with constant resistance, thereby improving respiratory efficiency, lung function, vital capacity, and maximum inspiratory pressure. This leads to increased cardiac output, venous return, and systemic blood flow. improved hemodynamics can facilitate the transport and clearance of metabolic waste products through the dialysis membrane, potentially enhancing dialysis adequacy [47]. Although Pellizzaro did not report statistically significant effects of IRME on dialysis adequacy [32], the network meta-analysis showed that IRME was associated with a statistically significant improvement in Kt/V compared to RHN. However, the effect size was small (Kt/V 0.09, 95% CI 0.03 to 0.16). This discrepancy may be due to Pellizzaro’s small sample size, which is more susceptible to random variation. In contrast, the network meta-analysis pooled data from multiple studies, thereby increasing the sample size and statistical power, and thus enabling the detection of smaller effects.

However, the results of this study showed that no statistically significant differences were observed between IAE + IRE, IAE, IRE, and IEMS compared with RHN. This outcome may be attributed to several factors. The variation range of Kt/V and URR is relatively small, and the improvement in dialysis adequacy through exercise intervention tends to be a gradual and cumulative process, making it difficult for short-term interventions to produce significant numerical changes. Moreover, many patients already had baseline dialysis adequacy close to the target, leaving limited room for further improvement. Additionally, non-intradialytic exercise interventions often lack standardized management and controllability. When patients exercise at home or in other environments, variations in exercise intensity, frequency, and adherence may introduce further heterogeneity into the study results. Therefore, SUCRA rankings were used to explore the potential trends of benefit among different intradialytic exercise modalities. The rankings consistently showed that all intradialytic exercise types had a higher probability of benefit than RHN, suggesting that intradialytic exercise may positively affect dialysis adequacy. However, further validation is needed in future studies.

The strength of this study lies in its focus on intradialytic exercise, particularly the inclusion of the novel IRME modality, which expands the range of exercise types and addresses existing gaps in the literature. By using both Kt/V and URR as outcome measures to assess dialysis adequacy, our study provides a more comprehensive evaluation compared to network meta-analyses that rely on a single outcome measure. This multi-dimensional approach enhances the interpretability and robustness of our findings.

The limitations of this study include the relatively small sample sizes for IEMS and IRME, which may have introduced bias in the overall effectiveness rankings. Although the meta-analysis only included randomized controlled trials, factors such as exercise intensity, frequency, and duration in each study may not have been adequately accounted for. Most studies had short-term follow-up, so it remains unclear whether the effects of these interventions would change over time. Furthermore, this study was based solely on published English-language literature, which may introduce regional bias and limit the generalizability of the findings.

## Conclusions

Current evidence is insufficient to conclude that any specific type of intradialytic exercise significantly improves dialysis adequacy. However, SUCRA rankings suggest a potential benefit, with IAE + IRE showing the highest likelihood of improvement. It is recommended that healthcare professionals in hemodialysis centers prioritize the implementation of the IAE + IRE intervention plan. For patients unable to perform active IRE or IAE, a passive exercise modality, IEMS, provides an alternative that may enhance dialysis adequacy. Future research should further investigate the impact of exercise during non-dialysis periods on dialysis adequacy. Additionally, high-quality randomized controlled trials on IEMS and IRME are needed to provide more substantial evidence for clinical practice and inform the development of relevant guidelines.

## Electronic Supplementary Material

Below is the link to the electronic supplementary material.

Supplementary Material 1

## Data Availability

No datasets were generated or analysed during the current study.
