# Supplementary material for: The efficacy of different types of intradialytic exercise for patients undergoing hemodialysis: a systematic review and network meta-analysis of randomized controlled trials
Source: BMC Nephrol. 2025 Aug 11;26:450. doi: 10.1186/s12882-025-04381-z (PMC12341117; doi:10.1186/s12882-025-04381-z)
Supplement: Supplementary file 1 — Supplementary Material 1 [file 12882_2025_4381_MOESM1_ESM.docx]

**Supplementary Material**

**Appendix 1.|** The search strategy in all databases.

**Table 1 Search strategy in PubMed-** **Retrieved from build to 1 November 2023**

| Search Number | Query | Results |
| --- | --- | --- |
| 6 | (#1 OR #2) AND (#3 OR #4) AND #5 | 1253 |
| 5 | ‘Dialysis adequacy’[Title/Abstract] OR ‘Kt/V’[Title/Abstract] OR ‘Urea Removal Index’[Title/Abstract] OR ‘urea clearance’[Title/Abstract] OR adequacy[Title/Abstract] OR efficiency[Title/Abstract] OR ‘solute removal’[Title/Abstract] OR quality[Title/Abstract] OR effects[Title/Abstract] OR solutions[Title/Abstract] OR urea[Title/Abstract] OR ‘solute clearance’[Title/Abstract] | 5992343 |
| 4 | Exercise[Title/Abstract] OR ‘physical workout’[Title/Abstract] OR ‘physical work-out’[Title/Abstract] OR ‘physical exertion’[Title/Abstract] OR ‘physical effort’[Title/Abstract] OR ‘physical conditioning, human’[Title/Abstract] OR ‘fitness workout’[Title/Abstract] OR ‘fitness training’[Title/Abstract] OR exertion[Title/Abstract] OR ‘exercise training’[Title/Abstract] OR ‘exercise performance’[Title/Abstract] OR ‘exercise capacity’[Title/Abstract] OR effort[Title/Abstract] OR ‘biometric exercise’[Title/Abstract] OR ‘Acute Exercises’[Title/Abstract] OR ‘Acute Exercise’[Title/Abstract] OR ‘Physical Activity’[Title/Abstract] OR ‘Physical Exercises’[Title/Abstract] OR ‘Exercise Trainings’[Title/Abstract] OR ‘Physical Activities’[Title/Abstract] OR ‘Aerobic Exercise’[Title/Abstract] OR ‘Isometric Exercise’[Title/Abstract] OR ‘Physical Exercise’[Title/Abstract] OR Exercises[Title/Abstract] | 643769 |
| 3 | Exercise[MeSH Terms] | 250994 |
| 2 | ‘renal dialysis’[Title/Abstract] OR ‘intermittent hemodialysis’[Title/Abstract] OR ‘intermittent chronic hemodialysis’[Title/Abstract] OR ‘intermittent chronic hemodialysis’[Title/Abstract] OR ‘intermittent hemodialysis’[Title/Abstract] OR ‘hospital hemodialysis units’[Title/Abstract] OR ‘hospital haemodialysis units’[Title/Abstract] OR hemotrialysate[Title/Abstract] OR hemorenodialysis[Title/Abstract] OR ‘hemodialysis unit’[Title/Abstract] OR ‘hemodialysis department’[Title/Abstract] OR ‘hemodialysis center’[Title/Abstract] OR hemodialyse[Title/Abstract] OR ‘haemodialysis unit’[Title/Abstract] OR ‘haemodialysis department’[Title/Abstract] OR ‘haemodialysis Centre’[Title/Abstract] OR ‘haemodialysis center’[Title/Abstract] OR haemodialysis[Title/Abstract] OR ‘extracorporeal dialysis’[Title/Abstract] OR ‘extracorporeal blood cleansing’[Title/Abstract] OR ‘dialysis center’[Title/Abstract] OR ‘chronic intermittent hemodialysis’[Title/Abstract] OR ‘chronic intermittent hemodialysis’[Title/Abstract] OR ‘chronic hemodialysis’[Title/Abstract] OR ‘chronic haemodialysis’[Title/Abstract] OR ‘blood dialysis’[Title/Abstract] OR ‘Extracorporeal Dialysis’[Title/Abstract] OR ‘Extracorporeal Dialyses’[Title/Abstract] OR ‘Renal Dialyses’[Title/Abstract] OR Hemodialysis[Title/Abstract] OR Hemodialyses[Title/Abstract] | 92019 |
| 1 | Renal Dialysis[MeSH Terms] | 127372 |

**Search strategy in Embase-** **Retrieved from build to 1 November 2023**

| Search Number | Query | Results |
| --- | --- | --- |
| #8 | #6 and #7 and #8 | 1790 |
| #7 | #3 or #4 | 977425 |
| #6 | #1 or #2 | 186954 |
| #5 | 'dialysis adequacy':ti,ab,kw OR 'Kt/V':ti,ab,kw OR 'urea removal index':ti,ab,kw OR 'urea clearance':ti,ab,kw OR adequacy:ti,ab,kw OR efficiency:ti,ab,kw OR 'solute removal':ti,ab,kw OR quality:ti,ab,kw OR effects:ti,ab,kw OR solutions:ti,ab,kw OR urea:ti,ab,kw OR 'solute clearance':ti,ab,kw | 7491715 |
| #4 | exercise:ti,ab,kw OR 'physical workout':ti,ab,kw OR 'physical work-out':ti,ab,kw OR exercises:ti,ab,kw OR 'physical exercise':ti,ab,kw OR 'physical exertion':ti,ab,kw OR 'physical effort':ti,ab,kw OR 'physical conditioning, human':ti,ab,kw OR 'fitness workout':ti,ab,kw OR 'physical exercises':ti,ab,kw OR 'fitness training':ti,ab,kw OR 'exercise trainings':ti,ab,kw OR 'biometric exercise':ti,ab,kw OR effort:ti,ab,kw OR 'exercise capacity':ti,ab,kw OR 'exercise performance':ti,ab,kw OR exertion:ti,ab,kw OR 'exercise training':ti,ab,kw OR 'acute exercises':ti,ab,kw OR 'acute exercise':ti,ab,kw OR 'physical activity':ti,ab,kw OR 'physical activities':ti,ab,kw OR 'isometric exercise':ti,ab,kw OR 'aerobic exercise':ti,ab,kw | 859920 |
| #3 | 'exercise'/exp | 452509 |
| #2 | 'renal dialysis':ti,ab,kw OR 'intermittent chronic hemodialysis':ti,ab,kw OR 'intermittent hemodialysis':ti,ab,kw OR 'hospital hemodialysis units':ti,ab,kw OR 'hospital haemodialysis units':ti,ab,kw OR hemotrialysate:ti,ab,kw OR hemorenodialysis:ti,ab,kw OR 'hemodialysis unit':ti,ab,kw OR 'hemodialysis department':ti,ab,kw OR 'hemodialysis center':ti,ab,kw OR hemodialyse:ti,ab,kw OR 'haemodialysis unit':ti,ab,kw OR 'haemodialysis department':ti,ab,kw OR 'extracorporeal dialyses':ti,ab,kw OR 'chronic haemodialysis':ti,ab,kw OR hemodialysis:ti,ab,kw OR 'extracorporeal blood cleansing':ti,ab,kw OR 'haemodialysis centre':ti,ab,kw OR 'haemodialysis center':ti,ab,kw OR 'renal dialyses':ti,ab,kw OR 'blood dialysis':ti,ab,kw OR 'extracorporeal dialysis':ti,ab,kw OR 'dialysis center':ti,ab,kw OR 'chronic intermittent hemodialysis':ti,ab,kw OR 'chronic hemodialysis':ti,ab,kw OR hemodialyses:ti,ab,kw OR haemodialysis:ti,ab,kw | 140725 |
| #1 | 'hemodialysis'/exp | 143172 |

**Search strategy in The Cochrane Library-** **Retrieved from build to 1 November 2023**

| Search Number | Query | Results |
| --- | --- | --- |
| #8 | #6 and #7 and #8 | 1443 |
| #7 | #3 or #4 | 217763 |
| #6 | #1 or #2 | 21200 |
| #5 | (‘Dialysis adequacy’ or ‘Urea Removal Index’ or ‘urea clearance’ or adequacy or efficiency or ‘solute removal’ or quality or effects or solutions or urea or ‘solute clearance’):ti,ab,kw | 1315468 |
| #4 | (Exercise or ‘physical workout’ or ‘physical work-out’ or ‘physical exertion’ or ‘physical effort’ or ‘physical conditioning, human’ or ‘fitness workout’ or ‘fitness training’ or exertion or ‘exercise training’ or ‘exercise performance’ or ‘exercise capacity’ or effort or ‘biometric exercise’ or ‘Acute Exercises’ or ‘Acute Exercise’ or ‘Physical Activity’ or ‘Physical Exercises’ or ‘Exercise Trainings’ or ‘Physical Activities’ or ‘Aerobic Exercise’ or ‘Isometric Exercise’ or ‘Physical Exercise’ or Exercises):ti,ab,kw | 214494 |
| #3 | MeSH descriptor: [Exercise] explode all trees | 38838 |
| #2 | hemodialysis units’ or ‘hospital haemodialysis units’ or hemotrialysate or hemorenodialysis or ‘hemodialysis unit’ or ‘hemodialysis department’ or ‘hemodialysis center’ or hemodialyse or ‘haemodialysis unit’ or ‘haemodialysis department’ or ‘haemodialysis Centre’ or ‘haemodialysis center’ or haemodialysis or ‘extracorporeal dialysis’ or ‘extracorporeal blood cleansing’ or ‘dialysis center’ or ‘chronic intermittent hemodialysis’ or ‘chronic intermittent hemodialysis’ or ‘chronic hemodialysis’ or ‘chronic haemodialysis’ or ‘blood dialysis’ or ‘Extracorporeal Dialysis’ or ‘Extracorporeal Dialyses’ or ‘Renal Dialyses’ or Hemodialysis or Hemodialyses):ti,ab,kw | 20888 |
| #1 | MeSH descriptor: [Renal Dialysis] explode all trees | 6603 |

**Search strategy in Web of Science-** **Retrieved from build to 1 November 2023**

| Search Number | Query | Results |
| --- | --- | --- |
| #4 | #1 AND #2 AND #3 | 5501 |
| #3 | TS=（Dialysis adequacy’ or ‘Urea Removal Index’ or ‘urea clearance’ or adequacy or efficiency or ‘solute removal’ or quality or effects or solutions or urea or ‘solute clearance’） | 878108 |
| #2 | TS=(Exercise or ‘physical workout’ or ‘physical work-out’ or ‘physical exertion’ or ‘physical effort’ or ‘physical conditioning, human’ or ‘fitness workout’ or ‘fitness training’ or exertion or ‘exercise training’ or ‘exercise performance’ or ‘exercise capacity’ or effort or ‘biometric exercise’ or ‘Acute Exercises’ or ‘Acute Exercise’ or ‘Physical Activity’ or ‘Physical Exercises’ or ‘Exercise Trainings’ or ‘Physical Activities’ or ‘Aerobic Exercise’ or ‘Isometric Exercise’ or ‘Physical Exercise’ or Exercises) | 66520 |
| #1 | TS=(‘renal dialysis’ or ‘intermittent hemodialysis’ or ‘intermittent chronic hemodialysis’ or ‘intermittent chronic hemodialysis’ or ‘intermittent hemodialysis’ or ‘hospital hemodialysis units’ or ‘hospital haemodialysis units’ or hemotrialysate or hemorenodialysis or ‘hemodialysis unit’ or ‘hemodialysis department’ or ‘hemodialysis center’ or hemodialyse or ‘haemodialysis unit’ or ‘haemodialysis department’ or ‘haemodialysis Centre’ or ‘haemodialysis center’ or haemodialysis or ‘extracorporeal dialysis’ or ‘extracorporeal blood cleansing’ or ‘dialysis center’ or ‘chronic intermittent hemodialysis’ or ‘chronic intermittent hemodialysis’ or ‘chronic hemodialysis’ or ‘chronic haemodialysis’ or ‘blood dialysis’ or ‘Extracorporeal Dialysis’ or ‘Extracorporeal Dialyses’ or ‘Renal Dialyses’ or Hemodialysis or Hemodialyses) | 92400 |

**Appendix 2 Table S1** – Characteristics of studies and subjects included in the review

| Author | Year of publication | No.of Patients (male) | | age[year,χ±s or M(Q)] | | type of interventions | | Exercise Content | Exercise Cycle | Exercise Intensity | Exercise Administrator | Dialysis adequacy |
| --- | --- | --- | --- | --- | --- | --- | --- | --- | --- | --- | --- | --- |
|  |  | C | I | C | I | C | I |  |  |  |  |  |
| Desai^18^ | 2019 | 21（15） | 13（10） | 70.1±13.0 | 64.6±16.6 | RHN | IAE | Perform bedside cycling exercises during the first 2 hours of dialysis treatment, each lasting 20-60 minutes. | Conduct the exercise three times per week for 4 months. | Moderate Intensity | / | Kt/V |
| Kim^19^ | 2022 | 21（10） | 18（10） | 57.76±12.32 | 57.61±13.69 | RHN | IAE | Perform bedside cycling exercises during the first 1 to 1.5 hours of dialysis treatment, each lasting 40-70 minutes. | Conduct the exercise three times per week for 3 months. | Moderate Intensity | Researcher | Kt/V |
| Malini^10^ | 2022 | 24（18） | 23（15） | 53.7±11.8 | 50.8±12 | RHN | IAE | Perform 30 minutes of simulated cycling exercise during dialysis treatment. | Conduct the exercise two times per week for 2 months. | / | Dialysis Nurse or Physical Therapist | Kt/V/URR |
| Groussard^20^ | 2015 | 10（8） | 8（5） | 68.4±3.7 | 66.5±4.6 | RHN | IAE | Perform 30 minutes of recumbent cycling training during the first 2 hours of dialysis treatment. | Conduct the exercise three times per week for 3 months. | Moderate Intensity | Professional | Kt/V |
| Zhao^21^ | 2020 | 15（8） | 15（10） | 41.53±11.73 | 44.48±15.12 | RHN | IAE | Perform 30 minutes of recumbent cycling training during the first 2 hours of dialysis treatment. | Conduct the exercise three times per week for 3 months. | Moderate Intensity | Professional | Kt/V/URR |
|  |  |  | 15（10） |  | 47.87±12.03 |  | IAE+IRE | Perform 30 minutes of recumbent cycling training during the first 2 hours of dialysis treatment, followed by 3-5 minutes of rest, and then 15 minutes of resistance training (such as using resistance bands). |  |  |  |  |
| Abreu^35^ | 2017 | 19（7） | 25（14） | 42.5±13.5 | 45.7±15.2 | RHN | IRE | Resistance training during dialysis involves using ankle weights and resistance bands in lower limb exercises. | Conduct the exercise three times per week for 3 months. | Moderate Intensity | Physical Therapist | Kt/V |
| Reboredo^22^ | 2010 | 11（4） | 11（4） | 43.5±12.8 | 49.6±10.6 | RHN | IAE | Perform 1 hour of recumbent magnetic cycling exercise during dialysis treatment. | Conduct the exercise three times per week for 3 months. | Low Intensity | / | Kt/V |
| Afshar^23^ | 2010 | 7（7） | 7（7） | 53±19.4 | 50.7±21.06 | RHN | IAE | Perform recumbent cycling exercises during the first 2 hours of dialysis treatment, lasting 10-30 minutes. | Conduct the exercise three times per week for 2 months. | Moderate Intensity | Physical Therapist or Doctor | Kt/V |
|  |  |  | 7（7） |  | 51±16.4 |  | IRE | During the first 2 hours of dialysis treatment, perform lower limb exercises using ankle weights, including knee flexion and extension and hip abduction and flexion. The exercises are divided into three sets, with 8-12 repetitions per set. |  |  |  |  |
| Dong^34^ | 2019 | 20（12） | 21（9） | 62.5（50.5-70.0） | 59（32.5-66.5） | RHN | IRE | During dialysis treatment, perform lower limb leg raise exercises using ankle weights and upper limb compression exercises using a resistance ball. The training intensity gradually increases to 5 kilograms for each leg, lasting 1-2 hours. | Conduct the exercise three times per week for 3 months. | Moderate Intensity | Rehabilitation Team | Kt/V |
| Martins^37^ | 2020 | 12（8） | 12（5） | 60.4±10.6 | 49.3±12.4 | IRE | IAE+IRE | During the first 2 hours of dialysis treatment, patients in the control group perform lower limb stretching exercises (simulated cycling and flexion-extension exercises of the upper and lower limb joints). In the observation group, in addition to the control group exercises, patients perform seated or lying exercises using ankle weights and dumbbells, with 10 repetitions per set and three sets for each exercise. | Conduct the exercise three times per week for 3 months. | Low Intensity | Research Team | Kt/V |
| Kopple^24^ | 2007 | 14（9） | 10（6） | 41.3±3.3 | 45.9±4.1 | RHN | IAE | During dialysis treatment, use a stationary cycling device for 30-60 minutes per session. | Conduct the exercise three times per week for 6 months. | Moderate Intensity | Research Team | Kt/V |
| Fernandes^25^ | 2019 | 19（/） | 20（/） | 42.63±11.16 | 44.25±11.30 | RHN | IAE | Start 1 hour after the beginning of dialysis treatment, and perform recumbent cycling training for 40 minutes per session. | Conduct the exercise three times per week for 2 months. | Moderate Intensity | Research Team | Kt/V |
| Roxo^38^ | 2016 | 20（11） | 20（9） | 54.65±19.93 | 46.40±15.43 | RHN | IEMS | During dialysis treatment, perform electrical stimulation on the patient's bilateral quadriceps muscles for 30 minutes per session. The electrical stimulation parameters are set to a pulse width of 350 microseconds, a frequency of 50 Hz, and the current intensity is adjusted according to the patient's tolerance. | Conduct the exercise three times per week for 2 months. | / | Research Team | Kt/V |
| Frey^26^ | 1999 | 6（3） | 5（3） | 53±13 | 40±11 | RHN | IAE | During the first 2 hours of dialysis treatment, perform 45 minutes of recumbent cycling exercise. | Conduct the exercise three times per week for 2 months. | Moderate Intensity | Research Team | Kt/V |
| Huang^39^ | 2020 | 16（11） | 16（12） | 37.63±10.31 | 43.81±10.25 | RHN | IAE+IRE | During the first 2 hours of dialysis treatment, perform 30 minutes of exercise by adjusting the resistance on a recumbent bike. | Conduct the exercise three times per week for 6 months. | Moderate Intensity | Research Team | Kt/V |
| Vogiatzaki^31^ | 2022 | 12（7） | 12（8） | 57.4±13.7 | 58.1±14.3 | RHN | IAE | During the first 2 hours of dialysis treatment, perform 60 minutes of exercise using a recumbent bike. | Conduct the exercise three times per week for 6 months. | Moderate Intensity | Nurse or professional fitness coach | Kt/V/URR |
| Cheema^33^ | 2007 | 25（17） | 24（17） | 65±12.9 | 60.0±15.3 | RHN | IRE | During dialysis treatment, each training session consists of two sets of eight repetitions, using dumbbells and ankle weights. | Conduct the exercise three times per week for 3 months. | High Intensity | Exercise Physiologist | Kt/V |
| Dobsak^12^ | 2012 | 10（17） | 11（17） | 60.1±8.2 | 58.2±7.2 | RHN | IAE | During the 2nd to 3rd hour of dialysis treatment, perform 40 minutes of exercise using a recumbent bike. | Conduct the exercise three times per week for 5 months. | Moderate Intensity | Doctors, nurses, and physical therapists | Kt/V/URR |
|  |  |  | 11（17） |  | 64.5±8.1 |  | IEMS | During the 2nd to 3rd hour of dialysis treatment, perform low-frequency electrical stimulation (EMS) on the patient's leg extensor muscles for 60 minutes. The EMS parameters are set as follows: frequency of 10 Hz, pulse width of 200 milliseconds, stimulation for 20 seconds, and rest for 20 seconds. | Conduct the exercise three times per week for 5 months. | Low-Frequency Intensity |  |  |
| Liao^27^ | 2016 | 20（9） | 20（8） | 62±9 | 62±8 | RHN | IAE | During the first 2 hours of dialysis treatment, perform 30 minutes of recumbent cycling exercise. | Conduct the exercise three times per week for 3 months. | Moderate Intensity | Doctor and Nurse | Kt/V |
| Makhloug^28^ | 2013 | 24（13） | 23（17） | 56±11 | 53±14 | RHN | IAE | During the first 2 hours of dialysis treatment, perform wrist, elbow, and ankle rotation and flexion-extension exercises for 15 minutes. | Conduct the exercise three times per week for 2 months. | Moderate Intensity | Doctor | Kt/V/URR |
| Oliveira^29^ | 2019 | 15（8） | 15（7） | 58±15 | 50±17.2 | RHN | IAE | During the first 2 hours of dialysis treatment, perform 30 minutes of recumbent cycling exercise. | Conduct the exercise three times per week for 4 months. | Moderate Intensity | Research Team | Kt/V |
| Tayebi^36^ | 2019 | 14（7） | 17（12） | 63.2±11.6 | 64.4±8.4 | RHN | IRE | Starting 30 minutes after the beginning of dialysis treatment, perform handgrip exercises and leg raise exercises. For the leg raise exercises, patients raise both legs while lying in bed, holding the position for 10 seconds. This is repeated for 10-15 sets. | Conduct the exercise two times per week for 2 months. | Moderate Intensity | Doctor | Kt/V/URR |
| Parsons^30^ | 2004 | 7（4） | 6（3） | 49±25 | 60±17 | RHN | IAE | During the first 3 hours of dialysis treatment, perform 15 minutes of cycling ergometer exercise in each session. | Conduct the exercise three times per week for 2 months. | Moderate Intensity | Research Team | Kt/V |
| Pellizzaro^32^ | 2013 | 14（8） | 11（8） | 51.9±11.6 | 43±13.8 | RHN | IRE | During the first 2 hours of dialysis treatment, perform knee extension exercises using leg weights. Complete three sets, each with 15 repetitions, with a 60-second rest between sets. | Conduct the exercise three times per week for 2.5 months. | Moderate Intensity | Research Team | Kt/V |
|  |  |  | 14（7） |  | 48.9±10.1 |  | IRME | During the first 2 hours of dialysis treatment, respiratory training is performed using the Threshold Load device, with three sets of 15 inhalations each, and a 60-second rest between sets. |  |  |  |  |

Abbreviations: including intradialytic aerobic exercise， IAE； intradialytic resistance exercise， IRE； Intradialytic electrical muscles stimulation， IEMS； Intradialytic respiratory muscle training， IRME； intradialytic aerobic and resistance mixed exercise ，IAE+IRE； routine hemodialysis nurse ，RHN；single pool clearance index， Kt/V； urea reduction ratio， URR
